# Unveiling the Dance of Crystals: A Surgical Odyssey in the Open Excision of Synovial Chondromatosis in the Right Knee

**DOI:** 10.7759/cureus.56901

**Published:** 2024-03-25

**Authors:** Saksham Goyal, Sandeep Shrivastav, Ratnakar Ambade, Aditya Pundkar, Ashutosh Lohiya, Suhit Naseri

**Affiliations:** 1 Department of Orthopaedics, Jawaharlal Nehru Medical College, Datta Meghe Institute of Higher Education and Research, Wardha, IND; 2 Pathology, Jawaharlal Nehru Medical College, Datta Meghe Institute of Higher Education and Research, Wardha, IND

**Keywords:** arthroscopy, arthrotomy, open surgery, chondromatosis, synovial

## Abstract

Within the synovial membrane, cartilaginous nodules form as a result of a relatively rare joint condition called synovial chondromatosis. This case study describes the open surgical treatment of a male patient, age 25, who had severe discomfort in his right knee. The patient had synovial chondromatosis. The choice for open surgery was made because of the large and difficult nature of the lesions, even though arthroscopic procedures are commonly used in the management of this problem. The patient's history included a restricted range of motion, edema, and chronic right knee discomfort. Multiple intra-articular loose bodies were discovered during the clinical examination and imaging examinations, which led to the decision to do surgery. Owing to the size and position of the chondromatous lesions, an open surgical technique was considered suitable. Given the favorable result in this young adult patient, open surgical management of synovial chondromatosis may be an effective treatment option, especially in cases with complicated or widespread involvement.

## Introduction

Synovial chondromatosis is a rare benign disorder characterized by the growth of cartilaginous nodules inside joint synovial membranes, most typically affecting major joints like the knee [1,2]. Although arthroscopic excision has historically been the best course of action for treating this illness, open surgical intervention may be required in certain cases, particularly in cases with difficult or severe lesions [3]. This case study examines a 25-year-old male patient with right knee involvement who presented, was diagnosed, and had effective open surgical therapy of synovial chondromatosis.

The rare frequency and vague symptoms of synovial chondromatosis make diagnosis difficult. Patients frequently exhibit reduced range of motion, joint discomfort, and edema, which are symptoms of other more prevalent joint diseases [4]. Diagnostic imaging investigations, such as magnetic resonance imaging (MRI) and X-rays, are essential for locating distinctive loose bodies inside the synovial cavity [5]. Since arthroscopic excision is less intrusive and requires less recovery time, it has long been the recommended treatment for synovial chondromatosis. Nonetheless, there are circumstances in which open surgical care is selected, as in the example that is being discussed. The size, quantity, or location of chondromatous lesions may make this necessary, making an open approach more practical for direct visualization and excision.

The clinical presentation of the patient, the results of imaging tests, and the surgeon's expertise are all carefully taken into account while deciding on the best surgical procedure. Due to the infrequency of synovial chondromatosis and the paucity of research on open surgical procedures, it is critical to record and disseminate these instances to provide important insights into the treatment of this illness. The purpose of this case report is to highlight the efficacious use of open surgical surgery in synovial chondromatosis while underscoring the need for customized treatment plans. We aim to add to the body of knowledge and encourage more studies into improving the standards for choosing the best surgical strategy for the best possible patient outcomes by sharing the clinical course and results of our 25-year-old male patient.

## Case presentation

The patient, a 25-year-old male, came to Acharya Vinoba Bhave Rural Hospital with the main complaint of six months of continuous right knee discomfort, edema, and restricted range of motion. His symptoms grew worse despite conservative treatment, which included rest and analgesics, so more research was necessary. The patient also gave a history of previous operation in which the lesions were removed arthroscopically one year ago. The patient had pain relief for five to six months following which he started experiencing the symptoms.

**Figure 1:**
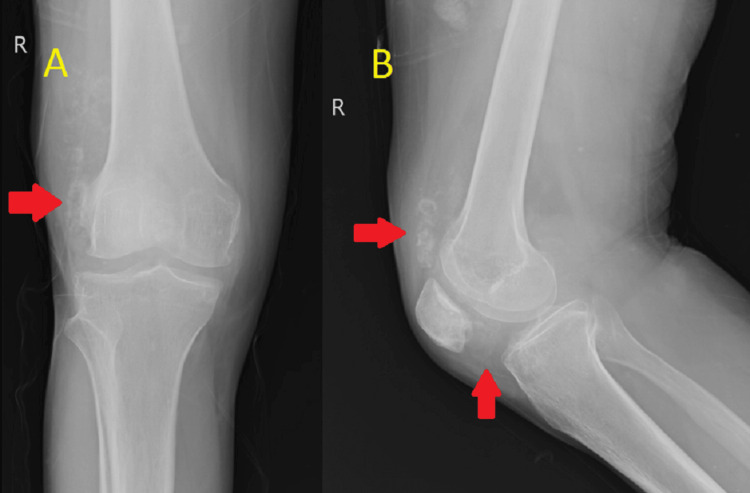
Anteroposterior (A) and lateral (B) radiographs of the knee showing multiple lesions like loose bodies Image A shows loose bodies as depicted by the arrow on the lateral aspect of the knee. Image B shows loose bodies in the suprapatellar and infrapatellar region

**Figure 2:**
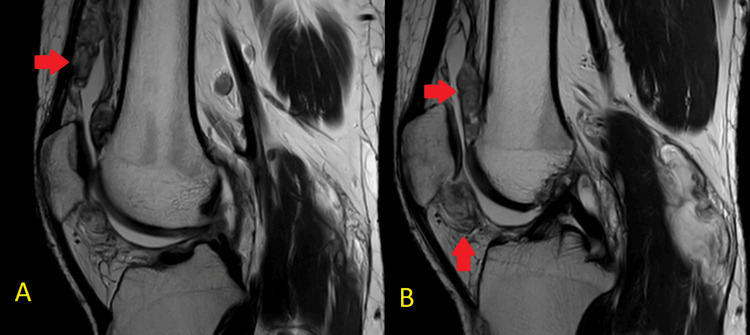
Sagittal MRI sections (A) and (B) showing multiple loose bodies T2-weighted images (A) and (B) of MRI show multiple loose bodies in the suprapatellar and infrapatellar region MRI: magnetic resonance imaging

**Figure 3:**
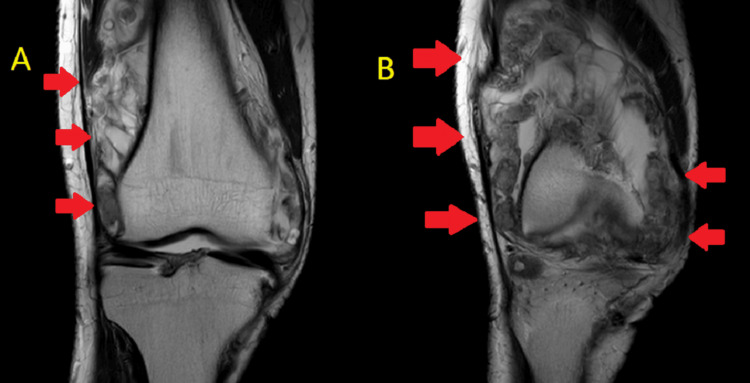
Coronal MRI sections (A) and (B) showing multiple loose bodies T2-weighted images (A) and (B) show multiple loose bodies circumferentially around the knee MRI: magnetic resonance imaging

**Figure 4:**
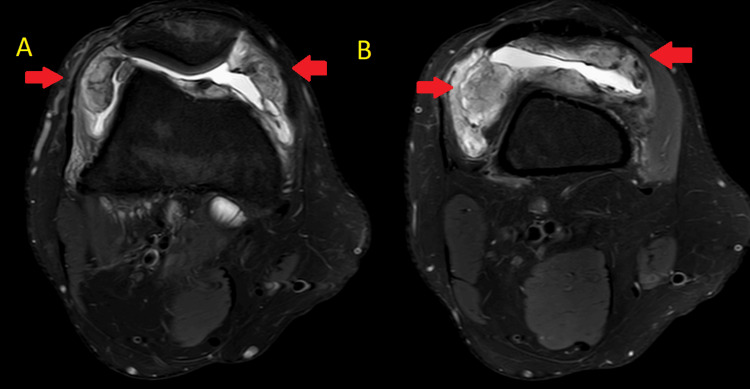
Axial MRI sections (A) and (B) showing multiple loose bodies T2-weighted images (A) and (B) show multiple loose bodies around the knee MRI: magnetic resonance imaging

Upon clinical examination, there was discomfort throughout the medial and lateral joint lines, palpable crepitus, and joint effusion. The patient reported intermittent locking and catching sensations within the knee. There were no systemic symptoms or other joint involvement. Initial imaging studies, including X-rays and MRI, indicated multiple intra-articular loose bodies and synovial thickening within the right knee joint, consistent with a diagnosis of synovial chondromatosis as shown in Figures 1-4.

The arthroscopic examination was initially considered for diagnostic confirmation and therapeutic intervention. However, given the extent and location of the chondromatous lesions observed on imaging, and after careful consideration of the patient's overall health, an open surgical approach was deemed more appropriate.

**Figure 5:**
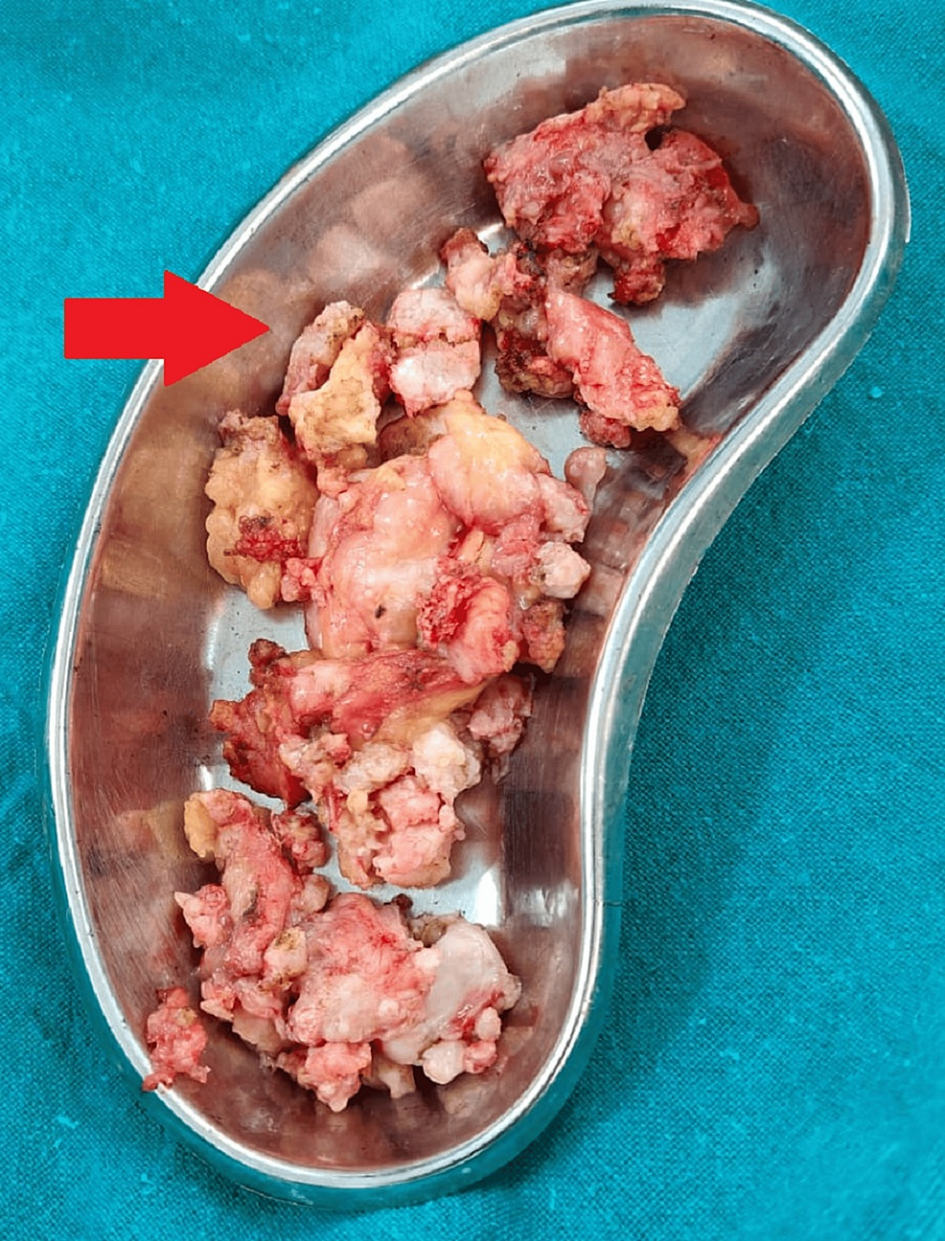
Surgically excised multiple synovial chondromatosis lesions

Under spinal anesthesia, the patient underwent an open arthrotomy of the right knee. A midline incision was made, and the joint was accessed through a medial parapatellar approach. Intraoperatively, the synovial membrane exhibited diffuse chondromatosis, with multiple cartilaginous nodules varying in size. These nodules were meticulously excised along with thorough synovectomy to remove affected synovial tissue as shown in Figure 5.

**Figure 6:**
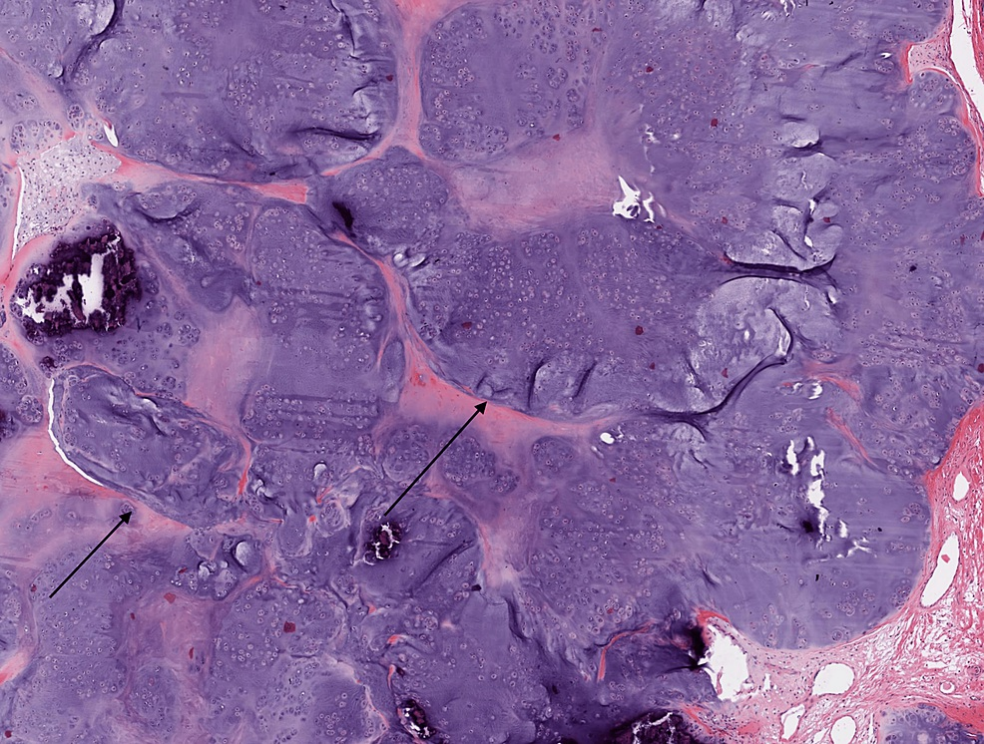
Microscopic image of the synovial chondromatosis Black arrows show multiple hyaline cartilage nodules with minimal atypia

Histopathological analysis of the excised specimens confirmed the presence of hyaline cartilage consistent with synovial chondromatosis, validating the preoperative diagnosis. No evidence of malignant transformation was identified. Microscopic image of the same is shown in Figure 6.

**Figure 7:**
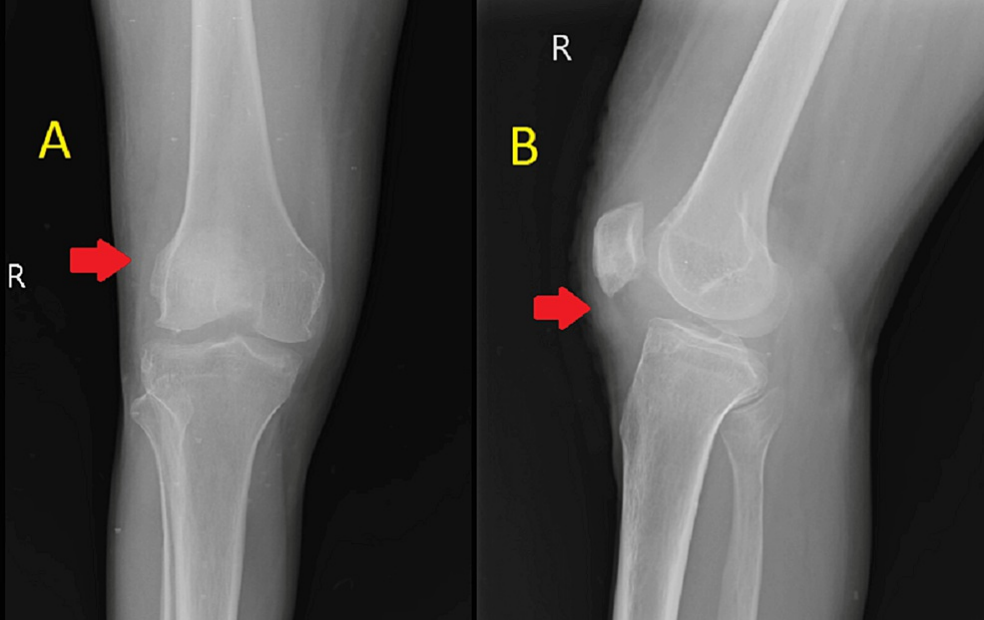
Postoperative radiograph anteroposterior (A) and lateral (B) views of the knee joint Images A and B show the successful excision of multiple loose bodies which were seen in preoperative radiograph at the same site depicted by arrows

The patient's postoperative course was uneventful. He was placed on a structured rehabilitation program to optimize joint mobility and strength. Pain and swelling significantly improved, and the patient reported a gradual return to normal activities. Follow-up examinations at three, six, and 12 months postoperatively demonstrated sustained improvement in knee function with no signs of recurrence. Postoperative radiograph is shown in Figure 7.

## Discussion

The etiology of synovial chondromatosis is uncertain, yet it may be caused by the metaplasia of synovial cartilage [6]. Sub-intimal fibroblasts in the tendons and bursae of synovial joints are impacted by the development of cartilaginous nodules or loose bodies in synovial tissues and joint cavities [7]. These nodules can protrude from the synovium and either go into the extra-articular soft tissue of joints or become loose bodies that float in the synovium [8]. The staging system may vary slightly among sources, but a common classification includes the following stages as depicted in Table 1.

**Table 1:** Staging of synovial chondromatosis

S. no.	Stage of synovial chondromatosis	Features
1	Stage I: active synovial lesions without loose bodies	In this early stage, there is an active synovial disease without the presence of loose bodies. Microscopic examination may reveal synovial cartilage metaplasia, where normal synovial tissue transforms into cartilage-like tissue.
2	Stage II: transitional stage with loose bodies within synovial proliferation	Transitional lesions are characterized by active intrasynovial proliferation. Free loose bodies may be identified within the proliferated synovial membrane. This stage involves a combination of ongoing synovial disease and the presence of loose bodies within the joint.
3	Stage III: multiple loose bodies released into the joint space	This advanced stage is marked by the release of multiple loose bodies into the joint space. Synovitis (inflammation of the synovial membrane) tends to subside at this point, but the presence of loose bodies can cause mechanical symptoms such as pain, swelling, and joint dysfunction [9].

It's crucial to remember that various sources may employ different standards or additional stages and that the staging technique may also change. Furthermore, there is variability in the clinical presentation of synovial chondromatosis, and there may not always be a regular staging pattern to the disease's course. Synovial chondromatosis typically manifests in patients between the ages of 30 and 50, while it can sometimes happen in children and adolescents [10,11]. Patients often present with joint pain, swelling, and restricted range of motion. The symptoms can mimic other joint pathologies, making an accurate diagnosis challenging. Advanced imaging modalities, including MRI and X-rays, play a crucial role in identifying characteristic loose bodies within the synovial cavity, aiding in the diagnosis [12]. Because of their great resolution of soft tissue, MRI scans are an effective means of identifying and characterizing synovial lesions [13,14].

For the treatment of synovial chondromatosis, arthroscopic excision is recognized as the gold standard. Direct visualization and removal of loose bodies are made possible by this minimally invasive technique while maintaining joint structures. Arthroscopy offers faster recovery periods and lower morbidity, making it especially beneficial for smaller joints and early-stage illness [15,16]. On the other hand, open surgical intervention can be necessary in situations with difficult or severe lesions. With direct access to the synovium made possible by this method, synovectomy and thorough excision are possible. The features of the lesion, the involvement of the joint, and the surgeon's experience should all be taken into consideration while deciding between arthroscopic and open surgery. With direct visualization of the whole joint made possible by the open arthrotomy technique, the surgeon may precisely excise the synovial chondromatosis. The best exposure is provided by the medial parapatellar incision, which makes it easier to remove many loose bodies and perform a thorough synovectomy.

Confirming the diagnosis and ruling out cancer requires a thorough histopathological examination of the excised specimen. Synovial chondromatosis was diagnosed due to the presence of hyaline cartilage in the nodules. It is comforting to see that there has been no malignant change, indicating that the surgical strategy used has been successful in managing the benign character of the illness. Recurrence is a potential concern in synovial chondromatosis, emphasizing the importance of meticulous excision and long-term follow-up. Even with effective initial treatment, chondromatous nodule recurrence may occur in certain individuals, requiring continued monitoring and, if necessary, further intervention. Although surgery is still the mainstay of treatment, other medical alternatives such as intra-articular corticosteroid injections and nonsteroidal anti-inflammatory drugs (NSAIDs) can also help reduce symptoms. These methods, however, are mostly supportive and do not deal with the underlying disease. While open surgical management proved successful in this case, it is essential to acknowledge potential challenges, including increased surgical invasiveness and the potential for prolonged recovery. Studies that compare the results of open and arthroscopic procedures may shed light on the best ways to treat synovial chondromatosis. The infrequency of synovial chondromatosis and the paucity of comparative studies comparing arthroscopic and open techniques underscore the necessity for more investigation to improve treatment algorithms and maximize results.

## Conclusions

The case study of a 25-year-old man with synovial chondromatosis highlights the effectiveness of open surgical care in some situations when the location and size of lesions make arthroscopic intervention difficult. The surgical team opted for a medial parapatellar approach, as guided by the thorough lesions shown on preoperative imaging, which supported the choice to undergo open surgery.

The management of synovial chondromatosis is improved by this case report, which highlights the significance of using a nuanced approach when choosing the best surgical procedure. The effective use of open surgery in this instance broadens our current understanding of the condition and motivates more study and discussion to improve our comprehension of and approaches to treating this uncommon joint ailment. To progress in the field and guarantee the best possible care for patients with synovial chondromatosis, collaboration and documenting of such instances must continue.
